# Species–specific circuitry of double cone photoreceptors in two avian retinas

**DOI:** 10.1038/s42003-024-06697-2

**Published:** 2024-08-14

**Authors:** Anja Günther, Silke Haverkamp, Stephan Irsen, Paul V. Watkins, Karin Dedek, Henrik Mouritsen, Kevin L. Briggman

**Affiliations:** 1 Department of Computational Neuroethology, Max Planck Institute for Neurobiology of Behavior—caesar, Bonn, Germany; 2 Electron Microscopy and Analytics, Max Planck Institute for Neurobiology of Behavior—caesar, Bonn, Germany; 3 Animal Navigation/Neurosensorics Group, Institute for Biology and Environmental Sciences, Carl von Ossietzky Universität Oldenburg, Carl-von-Ossietzky-Straße 9-11, Oldenburg, Germany; 4 Research Centre for Neurosensory Sciences, Carl von Ossietzky University of Oldenburg, Carl-von-Ossietzky-Straße 9-11, Oldenburg, Germany

**Keywords:** Neural circuits, Retina

## Abstract

In most avian retinas, double cones (consisting of a principal and accessory member) outnumber other photoreceptor types and have been associated with various functions, such as encoding luminance, sensing polarized light, and magnetoreception. However, their down-stream circuitry is poorly understood, particularly across bird species. Analysing species differences is important to understand changes in circuitry driven by ecological adaptations. We compare the ultrastructure of double cones and their postsynaptic bipolar cells between a night-migratory European robin and non-migratory chicken. We discover four previously unidentified bipolar cell types in the European robin retina, including midget-like bipolar cells mainly connected to one principal member. A downstream ganglion cell reveals a complete midget-like circuit similar to a circuit in the peripheral primate retina. Additionally, we identify a selective circuit transmitting information from a specific subset of accessory members. Our data highlight species-specific differences in double cone to bipolar cell connectivity, potentially reflecting ecological adaptations.

## Abstract (web summary)

Volume electron microscopy of retinas from two birds living in different habitats sheds light on species-specific wiring differences of double cone contacting bipolar cells and identifies principal and accessory member specific downstream circuits.

## Introduction

Birds, together with reptiles and amphibians, express the greatest diversity of retinal photoreceptor cell types among vertebrates (reviewed in ref. ^1^). In addition to rods, and four types of single cone photoreceptors that underlie (up to) tetrachromatic vision^2^, they possess double cone photoreceptors. Double cones are common to most tetrapods except eutherian mammals and may reflect an adaptation for vision in air^1^. They consist of a principal and accessory member both expressing a long-wavelength sensitive opsin^3,4^. Despite the prevalence of double cones (almost 50% of cones in birds^5–7^, their role in signal processing is not resolved, largely due to a lack of functional recordings and a description of their downstream synaptic connectivity. Identifying the functional role of double cones is crucial not only for understanding avian visual processing, but also for elucidating principles of visual sensory adaptation and evolution in vertebrates.

Currently, double cones have been suggested to perform achromatic functions such as encoding of luminance^8^, fine pattern recognition/high acuity vision^9^, or, as perhaps in some fish^10,11^, polarized light detection based on the observation of structured mosaics with a 90° orientation between neighbouring double cones in central regions of the retina^12^. Additionally, double cones have also been suggested to underlie light-dependent magnetoreception^13^ because, in many bird species, they express cryptochrome 4^4^. This blue-light sensitive protein was recently demonstrated to be magnetically sensitive via a light-dependent, radical-pair-based mechanism^14^. A specific orientation of cryptochromes within double cone members could in principle discriminate light intensity and polarization from magnetic field orientation, making the double cone a suitable site for a magnetoreceptive molecule. This hypothesis requires double cone members or neighbouring double cones to be oriented ~180° relative to each other^15^, an orientation recently demonstrated between neighbouring double cones in the peripheral avian retina^12^. Magnetic signals could then be potentially transmitted to bipolar cells that sample from differently oriented double cones. However, such bipolar cells have not yet been reported.

Initial evidence from chicken retinal ganglion cells suggests that birds use a complex and strikingly different strategy to encode visual stimuli than mammals^16^. This is consistent with findings using single cell transcriptomics, which revealed only a limited number of chicken ganglion cell types that are transcriptionally analogous to mammalian ganglion cells^17^. However, both studies focused on ganglion cells and lack information on the synaptic connectivity of upstream circuits, including the identification of bipolar cell types and their potential connectivity to double cones. Morphological studies on avian retinal neurons have been mainly performed in the chicken (summarised in ref. ^18^ or pigeon retina^19^). A detailed wiring diagram of bipolar cells in the chicken retina demonstrated that most bipolar cell types contact at least one member of the double cones, including an accessory member-selective type^7^. Whether this pattern of connectivity between double cones and bipolar cells generalises to other avian species is not known.

Detailed comparative mapping of the synaptic connectivity related to direction-selective circuits in mammals revealed that quantifying differences in wiring between species is crucial for distinguishing species-specific from more general properties of circuit function^20^. This highlights the need for comparative connectomic studies of retinal circuitry in avian species that occupy different ecological niches and migratory lifestyles.

In this study, we employ serial sectioning multi-beam scanning electron microscopy (ssmSEM) to generate detailed wiring diagrams of bipolar cell connectivity to double cones in the retina of the European robin, a long-distance night-migratory bird species adapted to hunting insects in flight. By comparing our findings with the previously studied retina of the ground foraging, non-migratory chicken^7^, we identified novel bipolar cell types and many differences in the postsynaptic connectivity of double cones, which could have a substantial impact on their circuit function. Furthermore, by focusing on downstream ganglion cells of either principal or accessory member dominated bipolar cell types, we identified retinal circuits dedicated to relaying double cone member-selective outputs to the brain.

## Methods

### Animals

A European robin with unknown sex, due to a lack of sexual dimorphism, was wild-caught near the University of Oldenburg using mist nets. Bird catching was done based on a permit issued by the Lower Saxony State Department for Waterway, Coastal and Nature Conservation (NLWKN, D7.2220/18). All animal procedures were performed in accordance with local, national and EU guidelines for the use of animals in research and were approved by the Animal Care and Use Committees of the Niedersächsisches Landesamt für Verbraucherschutz und Lebensmittelsicherheit (LAVES, Oldenburg, Germany).

### Sample preparation for electron microscopic recordings

A light-adapted European robin was killed by decapitation and the eyes were removed immediately. The lens apparatus and vitreous body were removed, and eyecups were fixed in 0.08 M cacodylate (Sigma Aldrich, St Louis, USA) buffer (pH = 7.4) containing 2.5% paraformaldehyde (Carl Roth, Karlsruhe, Germany) and 1.25% glutaraldehyde (EMS Hatfield, USA) for 30 min at room temperature. Retinas were removed from the eyecups and transferred into 0.08 M cacodylate buffer (pH = 7.4) two times for 15 min each.

The basic staining procedure was performed after^21^. Before embedding, the tissue was dehydrated through a graded ethanol series (50%, 75%, 100%, 30 min each at 4 °C), followed by washing three times in 100% anhydrous acetone (VWR, Radnor, USA) at room temperature for 30 min each. For epon infiltration, the tissue was first incubated in 1:2 mixture of anhydrous acetone and Embed812 resin hard formulation (20 ml Embed812, 9 ml DDSA, 12 ml NMA and 0.72 ml DMP-30; EMS) overnight at room temperature followed by 8 h incubation in pure Embed812 resin at room temperature. Retinas were cut into smaller pieces and the position within the retina was mapped before transferring the smaller tissue pieces into embedding moulds (Ted Pella) for polymerisation at 70 °C for 48 h.

### Sample sectioning and data acquisition for electron microscopy

After polymerization, a piece of the dorsal periphery in the left retina (for comparability, the European robin dataset was located in approximately the same region as a previously acquired chicken dataset in ref. ^7^) was pre-trimmed with a hand saw and afterwards trimmed to a block face of approximately 1 mm × 400 µm using a Leica ultramicrotome UC7. For the 3D dataset, serial sectioning of 40 nm thick slices was performed with a Diatome ultra ats diamond knife with a knife angle of 35° (Science Services, Germany) resulting in a volume depth of 36.4 µm. The cutting procedure was filmed to maintain slice order for recording and alignment. In total, 922 sections were collected on three glow discharged silicon wafers (Active Bizz, Germany) and dried on a heating plate at 50 °C until the water was fully evaporated. The wafers were mounted with silver paint (Plano) on a MultiSEM Universal holder Version 2 and stored in a heated vacuum chamber until further use. An overview image from each wafer was recorded with a Zeiss Axio Imager.A2 Vario. Individual slices were marked in the correct order using Zen2 (blue edition, Zeiss). The European robin sample was recorded with a 91-parallel-beam Zeiss MultiSEM 506. Recordings were performed with a beam current of 591 pA, landing energy of 1.5 keV, 400 ns pixel dwell time per beam and a pixel size of 4 nm. Image alignment was performed using custom written scientific python software, based on SIFT and normalised 2D cross correlation correspondences^22^. Skeletonization and synapse annotation were done in a smaller bounding box of 150 µm × 400 × 36.4 µm using the software webKnossos^23^. Subsequent analysis of skeletons and synapse annotations was performed using Matlab2023b. Reconstructed bipolar cells were grouped into complete and partial cells. Partial cells were defined as cells in which parts of the axon or dendritic field extended beyond the borders of the dataset. Completeness ranged from single truncated dendrites/axons to lacking the entire axonal/dendritic field. Only those partial bipolar cells were included in our analysis that could be clearly assigned to a cell type. From those, 194 bipolar cells were synapse annotated to map the connectivity to double cones.

The thickness of the inner plexiform layer (IPL) was calculated using webKnossos by measuring the length between the borders of the inner nuclear layer and the ganglion cell layer in single sections at 20 positions in the European robin and at 10 positions in the chicken dataset. Mean and standard deviation were calculated from these data.

### Analysis of photoreceptor cells and bipolar cells in a 3D volume

To calculate the prevalence of the different photoreceptor cell types, photoreceptors of the complete European robin dataset were analysed. Only photoreceptor cells which could be clearly identified as one specific type of photoreceptor were included in the statistical analysis. The identification of photoreceptor types was performed based on known morphological features, such as the presence or absence of an oil droplet in cones^24^, the presence of a paraboloid body in the inner segment of double cone accessory members^25^, and the localisation of the photoreceptor terminals in three strata of the outer plexiform layer^19^. We infer from the pigeon retina, that double cones and rods should be located in the first stratum whereas red/green single cones are in the second stratum and blue/UV cones in the third stratum. We also saw three strata in the outer plexiform layer in PSD95 stainings of the very peripheral European robin retina^26^. Additionally^19^, described straight axons for red and green cones and a bent axon for blue and UV (oblique) cones. In line with this, we also found that cones classified as green and red single cones based on terminal stratification, contain a straight axon whereas blue and UV cones have bent axons, shifting the terminal away from the soma. For comparability to the data published in ref. ^7^, long wavelength single cones (red cones) and middle wavelength single cones (green cones) as well as short wavelength single cones (blue cones) and ultra-short wavelength single cones (ultra-violet cones) were quantified in groups, respectively. For quantifying the number of synapses in a double cone terminal, we calculated the median number of basal and ribbon synaptic contacts in 5 exemplary double cones. Telodendria or bipolar cell dendrites that reached into the pedicle of a cone photoreceptor and ended there without contacting a ribbon synapse were classified as “basal contacts”. Telodendria or bipolar cell dendrites ending close to a terminal of a photoreceptor without making a contact to the pedicle were classified as “no clear contact”. Passing bipolar cell dendrites or photoreceptor telodendria that in the process contacted a terminal from a photoreceptor, were also classified as “no clear contact”. For evaluating the dendritic and axonal fields of the bipolar cells, we calculated the convex hull for each axonal and dendritic field using Matlab. Similar to our analysis in the chicken dataset^7^, bipolar cell types were classified primarily based on their axonal stratification profile in the IPL, which was divided into eight strata as described previously^7,27^. We calculated the pairwise correlation coefficients between the IPL stratification profiles of all bipolar cells using Matlab and found that cells that we classified into one cell type showed a high similarity within the resulting similarity matrix. Between-type similarities (off-diagonal boxes in the similarity matrix) arise from cell types that co-stratify in some strata of the IPL but not others. Additionally, the connectivity to photoreceptor cells and the formation of uniform mosaics as described in other species, such as mice^28,29^ and primates^30^, were further used to confirm cell type classifications. Lower similarity values of individual cells within a given cell type may be due to unusually small axonal fields within a stratum or slight shifts within the dataset. However, these cells could still be assigned based on the two additional criteria mentioned above.

### Calculation of double cone member orientation to each other

**Fig. 1:**
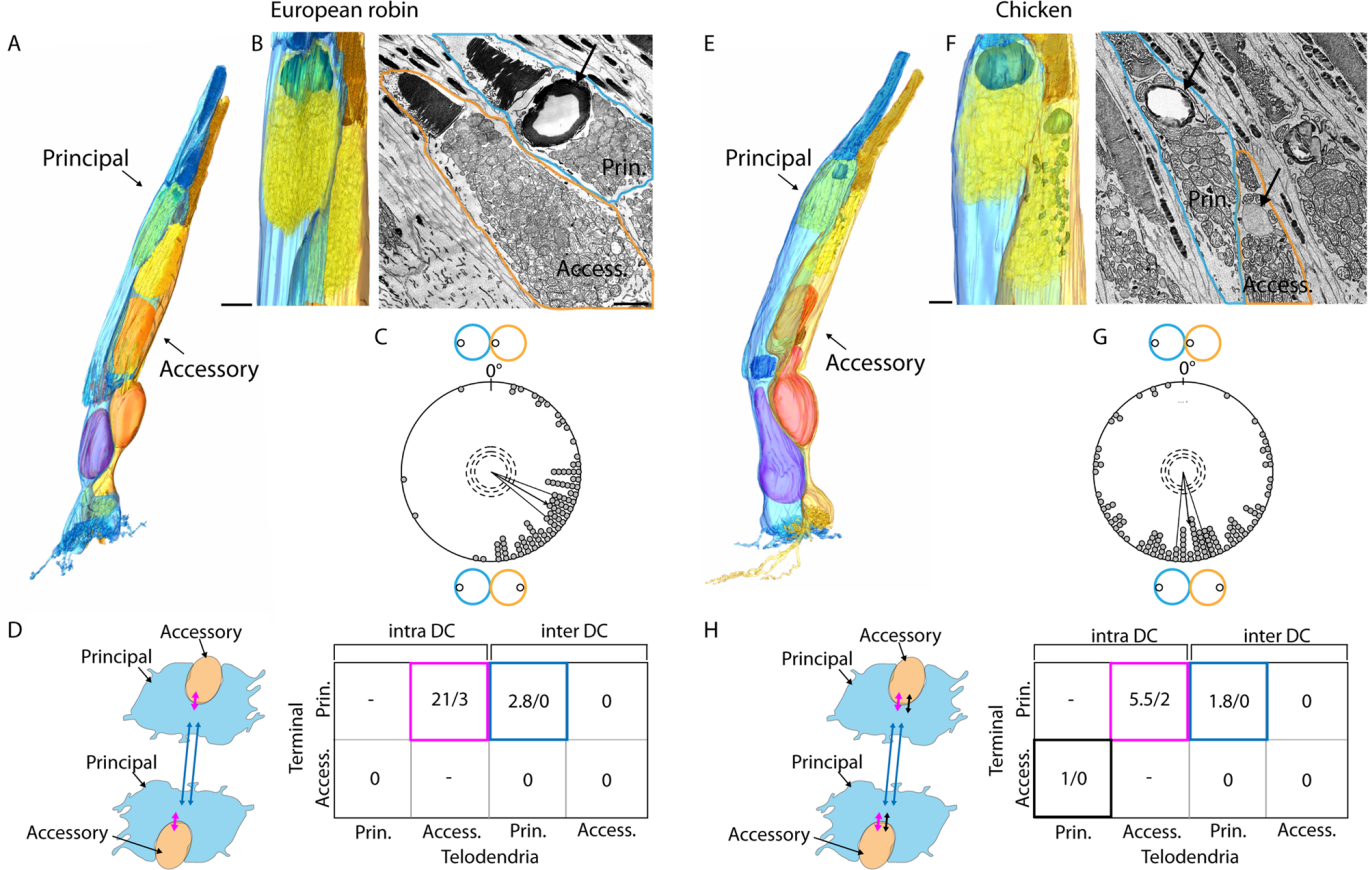
Double cone anatomy and connectivity in the European robin and chicken retina. **A**, **E** Volume reconstructions of a principal (blue) and accessory member (orange) of one double cone from the European robin and chicken, respectively. Volume reconstruction from the chicken double cone is taken from Günther et al. 2021. All major compartments (nucleus, mitochondria, Golgi-apparatus, oil droplet and paraboloid body in the accessory member) are highlighted in different colours. **B**, **F** Volume reconstructions and electron microscopic images of the inner segments of a European robin and chicken double cone, respectively. **C**, **G** Angles between principal and accessory member cilia within individual double cones in the European robin (*N* = 91) and chicken (*N* = 115), respectively. Grey dots represent angles between two cilia of one double cone in 5° bins. The arrows display the mean orientation of all double cones including the 95% confidence interval as solid lines. The arrow length represents the directedness based on their Rayleigh values (*r* value). Dashed lines indicate p levels (from inner circle to outer circle 0.05, 0.01, 0.001). Coloured circles represent outline of double cone member outer segments with cilia highlighted as black circles. **D**, **H** Schematic diagram of double cone connectivity and connectivity matrices showing the median number of intra and inter double cone basal/ribbon synaptic contacts between members (European robin *N* = 5, chicken *N* = 18). Directions of the arrows indicate the expected direction of signal transmission. Coloured arrows correspond to the respective colour coded numbers in the connectivity matrix. The double headed black arrow highlights the difference in principal member to accessory member connectivity between European robin and chicken. Prin principal member, Access accessory member, DC double cone. All scale bars: 1 µm.

Identifying the individual double cone member orientation is difficult given their almost cylindrical shape. We chose the connecting cilium between the inner and outer segment as a landmark to determine the individual cell orientation. Although the cilium is a robust landmark, it is not known whether other structures within the photoreceptors have fixed orientations relative to the cilium. The base of individual outer segments and the cilium contour were reconstructed in webKnossos. In a horizontal plane (schematically indicated in Fig. 1C) through the outer segments the cilium position appeared as a small circle within larger circles of outer segment outlines. The orientation of individual cells was calculated as the directional vector between the centroid of the outer segment outline and the centroid of the cilium. Intra-double cone orientation was then defined as the angle between the vectors of an accessory and principal member from one double cone. For inter-double cone orientation, the vector between the centroids of accessory and principal member from one double cone was calculated and the angle between double cone vectors was defined as inter-double cone orientation. The circular representation of the data as seen in Figs. 1C, G and 4B, D and Supplementary Fig. 2A was generated using a customised R script, including the calculation of statistical values (e.g., Rayleigh test) based on existing R functions from^31–33^.

### Stratification profile analysis of bipolar cells

To calculate the stratification profiles of bipolar cells in the IPL, we used a customized Matlab script to rotate the cells to horizontally align the axon terminals and equally interpolate the nodes within each skeleton with 100 nm spacing. Afterwards, we calculated the stratification profiles by summing the nodes across the IPL depth. To compare the plot profiles from the European robin with the chicken dataset published in ref. ^7^ which were based on volume reconstructions, we normalised the data from both species along the IPL depth.

### Analysis of B6a and B6b bipolar cell connectivity

Analysis of B6a and B6b bipolar cell type connectivity pattern to accessory member terminals was performed similarly to the double cone orientation. Direction vectors from accessory member outer segments were superimposed onto the terminal of the respective accessory member. The angle was calculated between the direction vector of the accessory member of a double cone and the local direction vector from the bipolar cell dendrite at the terminal.

### Reporting summary

Further information on research design is available in the Nature Portfolio Reporting Summary linked to this article.

## Results

### Photoreceptor cell type identification

We acquired a 1 mm × 400 µm × 36.4 µm high-resolution ssmSEM dataset from the dorsal periphery of an adult European robin retina (Supplementary Fig. 1) to analyse the distribution of photoreceptors and double cone specific circuits focusing only on bipolar cells postsynaptic to double cones. Identification of the photoreceptor cell type was performed based on known morphological features, such as the presence or absence of an oil droplet in cones^24^, and the presence of a paraboloid body in the inner segment of double cone accessory members^25^. We counted 610 photoreceptor cells among those 204 (33.4%) rods, 289 (47.4%) double cones and 117 (19.2%) single cones. Therefore, 71.2% of all cones were identified as double cones.

### Double cone anatomy and internal connectivity

We first reconstructed a European robin double cone including all of its major compartments (Fig. 1A) and compared it to a previously reconstructed chicken double cone (Fig. 1E^7^). Major differences were found such as the lack of long telodendria in the accessory member that reach out to contact single cone terminals (Fig. 1A) as observed in the chicken retina (Fig. 1E). Common for most vertebrate cones except in placental mammals, spectral properties are tuned by oil droplets located in the inner segments of cones^34,35^. In birds, the presence of an oil droplet in the accessory member seems to be species-dependent; it is present in chicken and pigeon^7,36^ but missing in turkey^37^ potentially resulting in differences in their spectral sensitivity. A previous study on European robins indicated regional variations in the presence of oil droplets in the accessory member^38^. In our analysis of the dorsal periphery, with one unclear case, the accessory member in the European robin lacks an oil droplet in the inner segment (*N* = 191) (Fig. 1B, Supplementary Data 1), while all accessory members in chicken contain oil droplets (Fig. 1F).

To investigate the orientation of double cone members necessary for some hypotheses regarding polarized light and/or magnetic field detection, we used the connecting cilium between inner and outer segments as a defining landmark to determine the orientation vector of individual principal and accessory members. The angle between the orientation vectors of the principal and accessory member within one double cone defined their relative orientation. We found that, in both chicken and European robin, the principal and accessory members of individual double cones exhibit opposing orientations with a mean relative orientation of 119.9° ± 46.1° in the European robin (Fig. 1C, *N* = 91, *R* = 0.72, *p* = <0.001) and 173.7° ± 60° in the chicken (Fig. 1G, *N* = 115, *R* = 0.58, *p* = <0.001). Additionally, neighbouring double cones were mostly ±180° to each other (Supplementary Fig. 2A) consistent with prior observations in peripheral retina^12^.

Double cones can form intra- and inter- synaptic contacts between both members (Fig. 1D, H). To characterize this connectivity, we differentiated between typical ribbon synaptic contacts and basal (flat) contacts^39,40^. We noted a clear difference between the ultrastructure of contacts formed by photoreceptor telodendria (originating from rods, single cones or double cones) compared to bipolar cell dendrites onto double cone pedicles (Supplementary Fig. 2B). For bipolar cell contacts, a clear cleft was observed between bipolar cell dendrites and double cone pedicles as described previously^41^. However, such a cleft was not observed between photoreceptor telodendria and double cone pedicles, indicating that photoreceptor telodendria may serve to form gap junctions between their axon terminals as previously described in the chicken retina^42^. To determine synapse densities within individual principal and accessory member terminals, we annotated ribbon and basal contacts in a subset of individual double cone members resulting in 423.2 ± 22.5 total contacts in principal member terminals (*N* = 5) and 102.2 ± 12 in accessory member terminals (*N* = 5). 11.1% of the contacts in European robin principal member terminals were formed by photoreceptor telodendria (of which 4.4% originated from rods, 3.9% from single cones, 49.1% from accessory members, 31.8% from neighbouring double cone principal members and 10.8% from unknown origin), while, with only one exception, there were no instances of any photoreceptor telodendria extending into the accessory member terminal. This contrasts with findings in the chicken (Fig. 1H, black arrows)^7^, in which the principal member forms intra-double cone contacts with the accessory member terminal, revealing a distinct asymmetry between the two species. Common to both bird species are the inter-double cone contacts between principal members (Fig. 1D, H; blue arrows). The median number of intra-double cone contacts from accessory member telodendria into the principal member terminal was substantially higher in the robin (21 basal + 3 ribbon contacts) than in the chicken (5.5 basal + 2 ribbon contacts) (Fig. 1D, H). We also found that telodendria from a double cone member occasionally contact their own cone pedicle, but there is currently no reported evidence for recurrent synaptic transmission within individual photoreceptors in the avian retina.

In summary, we identified striking differences in double cone anatomy and both intra- and inter-double cone connectivity between the chicken and European robin retinas, which could lead to species-specific differences in the response properties of double cones to visual stimuli.

### Bipolar cell type comparison

**Fig. 2:**
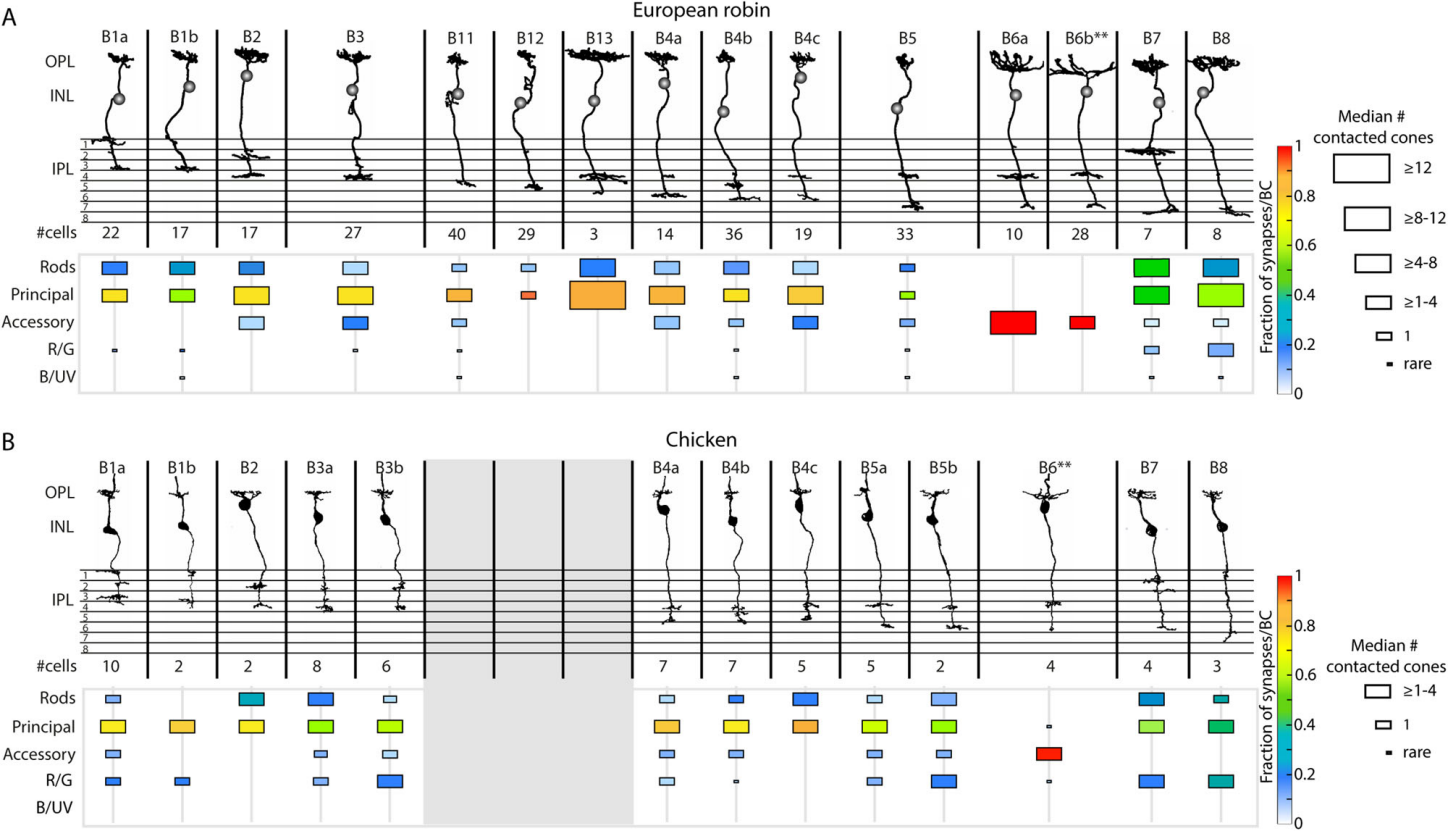
Species-specific bipolar cell types. **A**, **B** Bipolar cell types from the European robin and chicken retina, respectively, sorted based on their axonal stratification depth in the IPL. Single cone specific bipolar cells are not shown. Number of reconstructed cells per cell type is indicated below each type. Median number of contacted photoreceptor cells/cell type by different bipolar cell types are indicated by the size of the rectangles, and the colour indicates the fractionated number of combined basal and ribbon contacts per terminal per bipolar cell type. **B** modified from Günther et al. 2021. **Bipolar cell dendrites frequently extend beyond the limits of the dataset. OPL outer plexiform layer, INL inner nuclear layer, IPL inner plexiform layer, R/G red and green single cones, B/UV blue and ultraviolet single cones.

We next reconstructed all bipolar cells found to be postsynaptic to at least one double cone member within a sub-volume spanning approximately 150 × 400 × 36.4 µm. In total, we reconstructed 380 bipolar cells with somas located in the sub-volume. From those, 310 bipolar cells could be assigned to double cone contacting bipolar cell types, comprising 177 complete and 133 partial cells. The remaining 70 bipolar cells were partial cells that were either too close to the edge of the volume to be assigned to any type or did not contact the terminals of double cones and are presumed to be single cone bipolar cells. Based on our morphological classification approach (see Methods), we identified 15 bipolar cell types connected to European robin double cones. 210 bipolar cells correspond to bipolar cell types previously described in the chicken (Fig. 2B^7^) and 100 bipolar cells belonged to 4 novel types (Fig. 2A). The classical division into ON und OFF bipolar cells as in mammals, with OFF bipolar cells forming basal contacts with cone pedicles and terminating in the outer half of the inner plexiform layer (IPL) and ON bipolar cells forming ribbon contacts and terminating in the inner half of the IPL (reviewed in ref. ^43^), does not seem to apply to the bird retina. This is due to the multistratified nature of most avian bipolar cells combined with a less clear separation of ON and OFF layers in the IPL^7,26^ similar to what is known from the turtle^44^ and zebrafish^45^ retina.

**Fig. 3:**
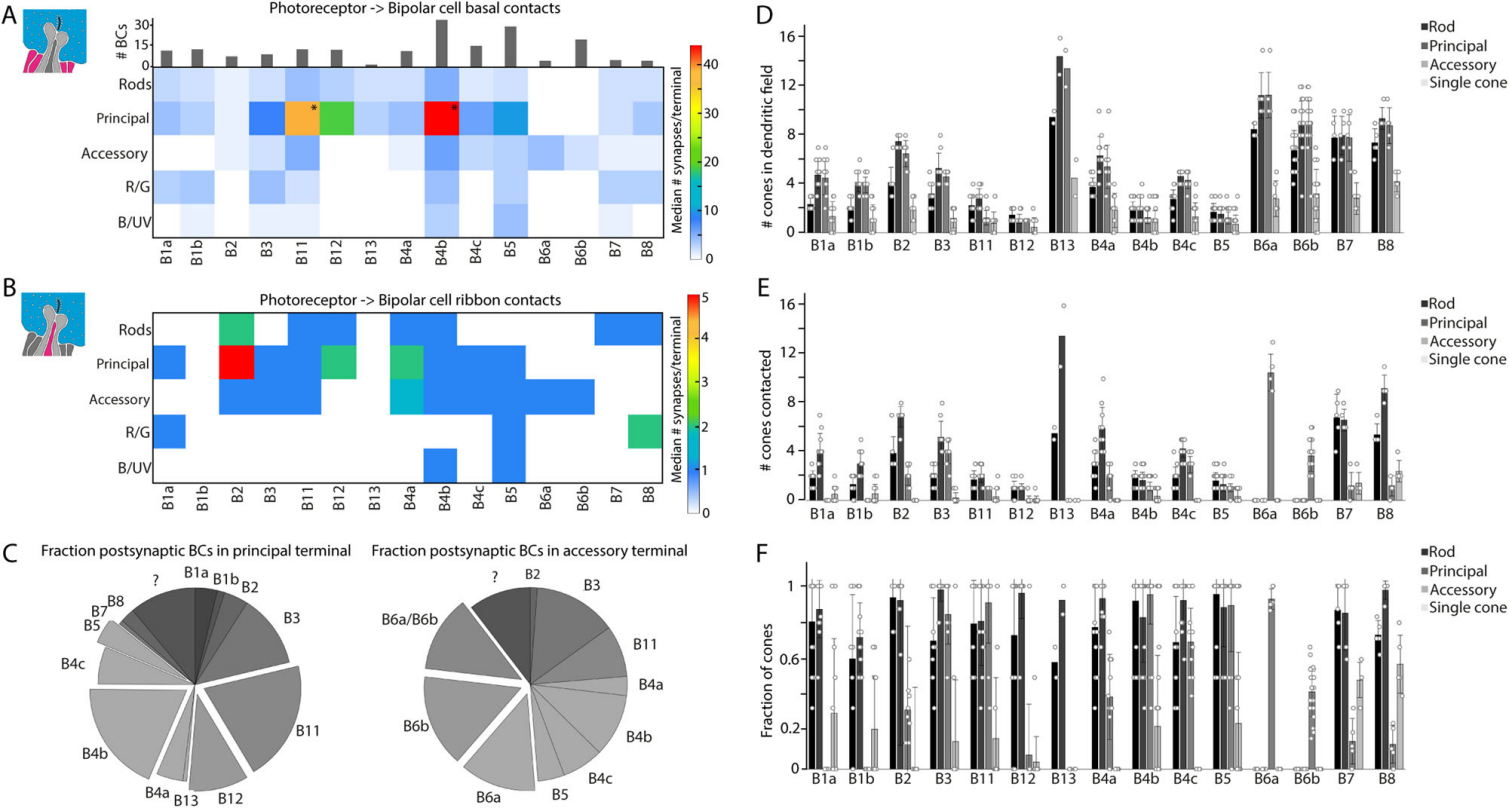
Quantification of photoreceptor to bipolar cell contacts in European robin. **A** Connectivity matrix with median number of basal contacts per bipolar cell type (magenta processes in the schematic drawings) in European robin retina. Histograms above the connectivity matrix indicate the number of cells that were included into the analysis. Two bipolar cell types showed a bimodal distribution of contacts between principal terminals in the centre and the periphery of the bipolar cell dendritic field (*). The number of basal contacts were thresholded only in those cases (all synapses >8/terminal) to show the number of contacts with the centre principal terminal. The complete connectivity matrix with the non-thresholded data at these two positions can be found in Supplementary Fig. 5. **B** Connectivity matrix with median number of ribbon synaptic contacts per bipolar cell type in European robin (magenta processes in the schematic drawings). No bimodal distribution was observed and therefore all data are non-thresholded. **C** Average fraction of synapses per bipolar cell type in individual principal or accessory terminals (*N* = 5). Some dendrites could clearly be assigned to accessory member-specific B6 cells but not subdivided into B6a or B6b and therefore were grouped into a separate B6a/B6b group. **D** Mean ± SD of cone terminals within the dendritic field of different bipolar cells. **E** Mean ± SD of contacted photoreceptor cells/type for different bipolar cell types. **F** Mean fraction ± SD of contacted/in dendritic field photoreceptor cells for different bipolar cell types. BC bipolar cell, R/G red and green single cones, B/UV blue and ultraviolet single cones.

Consistent with an earlier report^26^, we did not observe a Landolt’s club in any of the reconstructed robin bipolar cells, although Landolt’s clubs have been reported in several vertebrates, including chicken and pigeons^7,19,46,47^. For synapse annotation between bipolar cells and double cones, a subset of complete and partially reconstructed cells with complete dendritic fields was used (*N* = 194). The number of individual bipolar cells analysed for each cell type ranged from 2 to 35 (Fig. 3A).

**Fig. 4:**
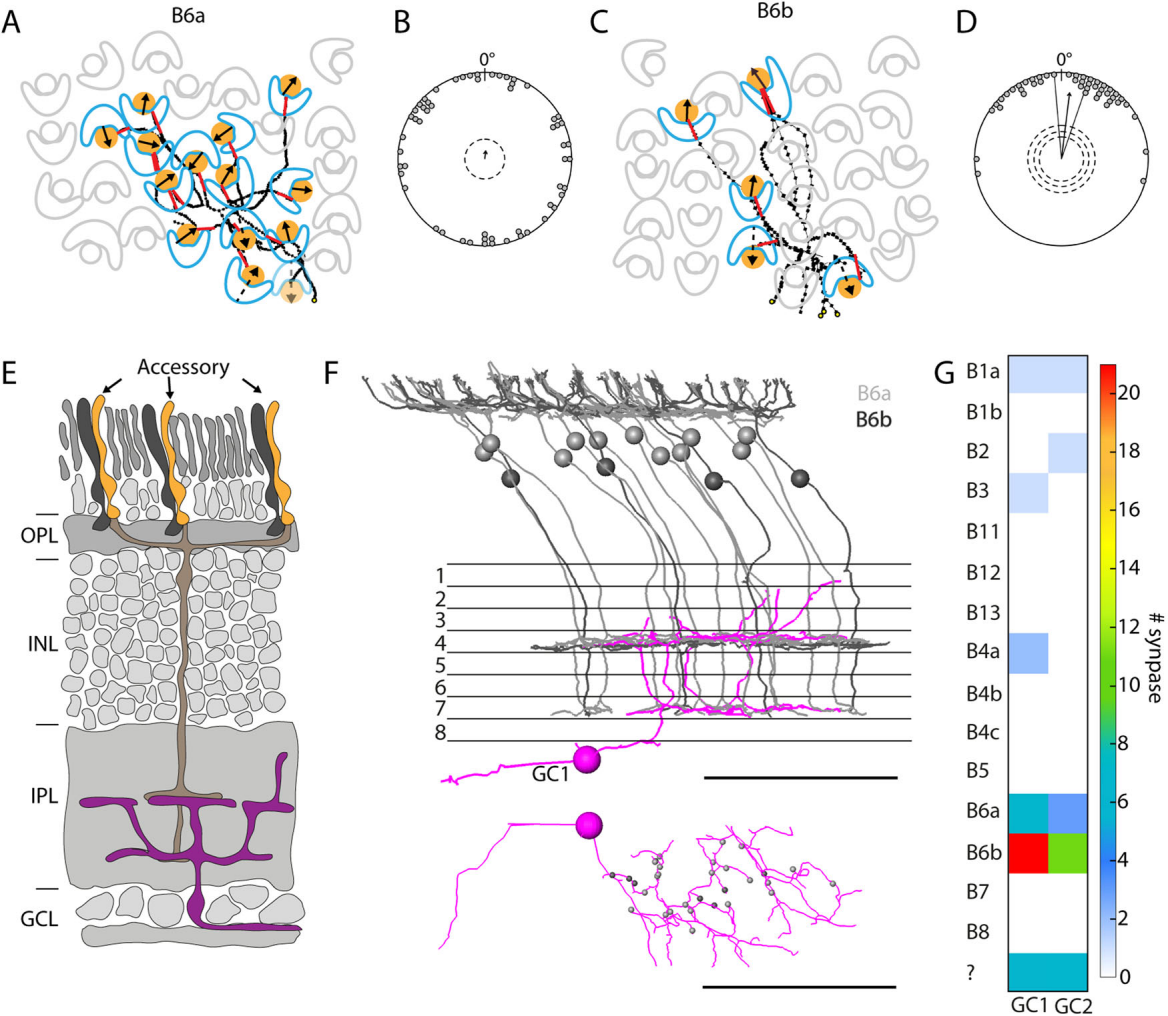
Accessory member specific circuit in the European robin retina. **A**, **C** Example dendritic fields highlighting the connectivity pattern of B6a and B6b bipolar cells, respectively. Only accessory members (orange circles) are contacted while principal members (blue boomerang) from the same double cones are not contacted. Arrows indicate accessory member orientation and red lines indicate the local dendritic bipolar cell orientation at the terminal used to calculate type specific connectivity patterns in (**B**) and (**D**). **B** Angle between local bipolar cell dendrite vectors and accessory member orientation vectors for B6a bipolar cells (# cells = 7, # dendrites = 57). **D** Angle between local bipolar cell dendrite vectors and accessory member orientation vectors for B6b bipolar cells (# cells=17, # dendrites = 46). Individual dots represent angle between one dendrite and an accessory member in 5° bins. The arrows display the mean angle of all dendrite- accessory member connections including the 95% confidence interval as solid line in case of significance. The arrow length represents the directedness based on their Rayleigh values (*r* value). Dashed lines indicate *p* levels (from inner circle to outer circle 0.05, 0.01, 0.001). **E** Schematic diagram of the B6a/B6b selective circuitry. OPL outer plexiform layer, INL inner nuclear layer, IPL inner plexiform layer, GCL ganglion cell layer. **F** Example of a B6a/B6b contacting ganglion cell with B6a and B6b cells shown in side view and individually from top view. Scale bars = 50 µm. **G** Connectivity matrix with number of synapses per bipolar cell type of two partially skeletonized B6a/B6b dominated ganglion cells with a total path length of approx. 1 mm.

To assess the similarity of bipolar cell connectivity between European robin and chicken retinas, we calculated the fractional number of synapses for each bipolar cell type in both species (Fig. 2A, B, colour in lower panels), including both basal and ribbon synaptic contacts. Additionally, we quantified the median number of photoreceptor terminals that bipolar cells of each type contact (Fig. 2A, B, proportional to the size of rectangles in lower panels). In many cases, European robin bipolar cell types match to those in chicken (including B1a, B1b, B2, B4a, B4b, B4c, B7 and B8) (Fig. 2). However, some individual types in the European robin correspond to two types in the chicken retina (B3 and B5) or a single type in the chicken corresponded to two bipolar cell types in the European robin (B6a and B6b). This direct comparison revealed that most of the bipolar cell types in both species receive the majority of their inputs from at least one principal member terminal (Fig. 2A, B). In contrast, the connectivity of bipolar cells to accessory members and red/green single cone photoreceptors varied substantially between both species, e.g., chicken B1a bipolar cells receive input from accessory members, but not in the European robin. Vice versa, European robin B2, B4c, B7 and B8 cells receive input from the accessory member, but not in chicken. The accessory member-selective B6 bipolar cells in the European robin formed two mosaics with slightly different stratification profiles, so we divided them into types B6a and B6b (Supplementary Fig. 4B, C), that ultimately were found to also have differences in their connectivity patterns (Fig. 4).

We identified four bipolar cell types in the European robin that were not previously described in the chicken: two narrow-field bipolar cells (B11 and B12) and two large-field bipolar cells (B13 and B6b). However, the absence of the two large-field bipolar cells in the chicken could be explained by the limited size of the dataset. In contrast, the two narrow-field bipolar cells, located directly below a principal member terminal, seem small enough to have been detected within the chicken dataset. Comparing the IPL stratification profiles of bipolar cell types across species, we observed a notable shift in the stratification profiles of robin bipolar cells beneath the layer where B11 and B12 stratify (Supplementary Figs. 3 and 4) providing further evidence of their absence in the chicken. Additionally, we measured the absolute IPL thickness in both species to investigate whether these two cell types led to an increase in the European robin. However, we found that the thickness in the European robin (52.5 ± 3.6 µm) is less than the chicken (56.2 ± 1.3 µm), potentially reflecting a more peripheral eccentricity of the European robin dataset compared to the chicken. In addition, a difference in retinal eccentricity between the two datasets might also explain some of the subtle changes in bipolar cell connectivity.

### Contacts between European robin bipolar cells and cones

To further investigate the convergent connectivity of photoreceptors onto the different bipolar cell types, we counted the median number of basal (Fig. 3A) and ribbon (Fig. 3B) synaptic contacts each bipolar cell receives per photoreceptor terminal of each type. We found that four types (B11, B12, B4b and B5) form a high number of basal and ribbon synaptic contacts to one principal member in the centre of their dendritic fields. B11 and B4b made a lower number of contacts to one or more additional principal member terminals in the periphery of their dendritic field resulting in a bimodal distribution of the synapse counts per terminal. To illustrate the high number of contacts formed specifically with the central principal member in these two bipolar cell types, we applied a threshold to the number of basal contacts, excluding terminals with <8 contacts before recalculating the median (Fig. 3A asterisks; see Supplementary Fig. 5 for the matrix without applied threshold). All other contacts within the connectivity matrix were not subject to thresholding. We also found that B2 bipolar cells are the only cells that primarily form ribbon contacts but stratify in the assumed OFF layers of the IPL (Fig. 3B).

We next evaluated the fractional divergent connectivity of individual principal and accessory member terminals onto the different types of bipolar cells (Fig. 3C). In general, all identified bipolar cells received input from the principal member, with the exception of B6a and B6b cells. The narrow-field bipolar cells (B11, B12, B4b and B5) account for nearly 50% of all synapses within the principal member terminal (Fig. 3C, highlighted wedges). In contrast, several bipolar cells (B1a, B1b, B7 and B8) avoid the accessory member terminal and the accessory member-specific B6a and B6b bipolar cells account for approximately 40% of all contacts (Fig. 3C, highlighted wedges).

We observed that most double cone-contacting bipolar cell types contact only a few single cone photoreceptors. To test whether these bipolar cell types avoid single cone terminals, we compared the number of photoreceptors within the dendritic field of a given bipolar cell (Fig. 3D) with the number that are synaptically connected (Fig. 3E) and calculated the ratio (Fig. 3F). For simplicity, we grouped all single cones into one category. In general, no bipolar cell contacted all of the photoreceptors within its dendritic field, indicating some degree of selectivity based on the photoreceptor type. For bipolar cell types contacting rods and principal members, 60–96% of these photoreceptor terminals were contacted within their dendritic fields, with some variation between the different bipolar cell types (Fig. 3F). In contrast, the variation for accessory members was much larger, ranging from 13% to 95%. We generally observed a low fraction of contacted single cones among all bipolar cells, ranging from 3–57%. Since we initially pooled all single cone types into one column, we further analysed whether this fraction changes between red/green and blue/ultraviolet single cones. With the exception of type B4b, bipolar cells that contacted single cones avoided blue/ultraviolet single cones (0–25%) and primarily contacted the red/green single cones within their dendritic fields (50–100%) (Supplementary Fig. 5).

### Accessory member-selective B6a and B6b bipolar cells in the European robin

During our analysis of bipolar cell connectivity, we noticed that B6b bipolar cells do not form contacts with all of the available accessory member terminals within their dendritic fields (Fig. 3D–F). To explore a potential underlying pattern, we overlaid the measured orientation of the accessory members with the bipolar cell dendritic trees (Fig. 4A, C). We found that B6a bipolar cells contacted nearly all accessory members within their dendritic field irrespective of their orientation (Fig. 4A, individual cells in Supplementary Fig. 6). Although it was only possible to partially reconstruct B6b cells due to their large dendritic field size, we found evidence that B6b bipolar cells only contact accessory members that have a specific orientation with respect to the postsynaptic dendrites (Fig. 4C, individual cells in Supplementary Fig. 7). We quantified this observation by calculating the angle between the accessory member orientation vector and the local vector representing the orientation of the dendrite before entering the terminal. Since B6a bipolar cells contacted almost all accessory members irrespective of their orientation, the angle between accessory members and dendrites was random (Fig. 4B, *N* = 7, dendrites = 57, *R* = 0.095, *p* = 0.59). B6b dendrites showed a strong preference to synapse with accessory members that are aligned with their local dendrites with a mean angle of 7° ± 41.4° (Fig. 4D, *N* = 17, dendrites = 46, *R* = 0.77, *p* < 0.001). Given this selectivity, we recalculated the fraction of contacted accessory members, but now including only those accessory members with the preferred orientation within the dendritic field (defined as accessory member orientation aligned ±45° to the local dendritic orientation). With this correction, B6b cells contact 68% (instead of 42% without correction) of all accessory members with their preferred orientation, indicating that this may not be the only defining factor for their selectivity. The extensive dendritic field of the B6b bipolar cells prevented us from fully reconstructing their entire dendritic fields, thereby limiting our ability to conduct further analyses, such as determining the overall distance between each contacted accessory member and the primary dendrite.

Only B6a and B6b cells exclusively contact one member of the double cone. To preserve this accessory member-selectivity in downstream circuits (Fig. 4E), the presence of a B6a and/or B6b selective ganglion cell would be required. We therefore reconstructed ganglion cells that were postsynaptic to B6a and B6b bipolar cells in the IPL. We identified two ganglion cells that were bistratified and received inputs predominantly from B6b and, with fewer inputs, from B6a in both strata (Fig. 4F, G). Although these reconstructed cells are only partial, it indicates that an accessory member-selective circuit likely exists in the European robin retina. Furthermore, type B5 axons co-stratify with B6a and B6b axons but were not synaptically connected to these ganglion cells.

### Narrow-field B12 bipolar cells predominantly receive input from one principal member

**Fig. 5:**
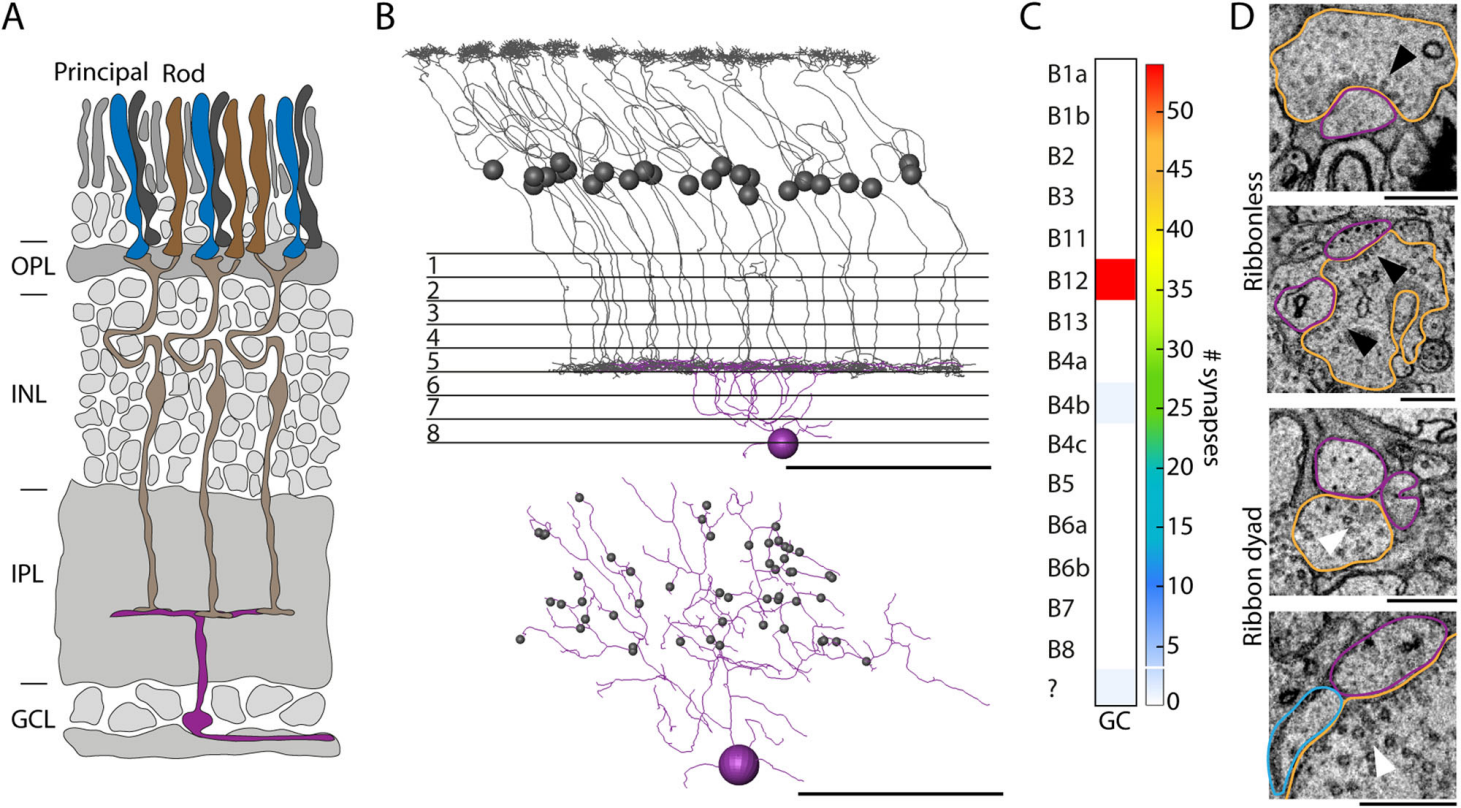
B12 specific circuit in the European robin retina. **A** Schematic diagram of a B12 specific circuit highlighting the predominant input from the principal member of the double cones to the B12 bipolar cell. OPL outer plexiform layer, INL inner nuclear layer, IPL inner plexiform layer, GCL ganglion cell layer. **B** Postsynaptic ganglion cell (purple) innervated by B12 bipolar cells shown in side view and individually from top view. Scale bars = 50 µm. **C** Connectivity matrix with number of synapses per bipolar cell type of one partial skeletonized B12 dominated ganglion cell with a path length of approx. 1 mm. **D** Electron microscopic images of ribbonless (black arrowheads) contacts and ribbon dyads (white arrowheads). Bipolar cell terminals are highlighted in orange, ganglion cell dendrites in magenta and potential amacrine cells in blue. Scale bars = 0.5 µm.

We identified two previously undescribed midget-like bipolar cells, B11 and B12, and observed an unexpected anatomical feature. The descending axons of B11 bipolar cells and the primary dendrites of B12 bipolar cells form one to several loops within the inner nuclear layer, extending the path length of the primary axon and dendrite by 72.6 ± 6.2 µm and 31.7 ± 7.6 µm, respectively (Figs. 2A and 5B). To a lesser extent, we also observed loops in the axons of B3 bipolar cells (14.8% of reconstructed B3 cells). B12 bipolar cells exhibit the strongest midget-like character by forming more than 90% of their contacts with only one principal member terminal (Fig. 2A) and contacting only one additional rod. To further investigate the circuitry of B12 bipolar cells, we searched for postsynaptic ganglion cell dendrites and found a ganglion cell with strong B12 input (Fig. 5B). 96.4% of all ribbon synapses onto this ganglion cell belonged to B12 bipolar cells (Fig. 5C). We found that the type of contact was either a classical ribbon dyad or a ribbon-less contact (Fig. 5D), as recently described for mouse and rabbit retina^48^. Since we cannot identify neurotransmitter receptors in our dataset, we cannot be sure that signal transmission occurs at the site of ribbonless contacts, but, at least in primates, ionotropic glutamate receptors are expressed at such sites^49^. Interestingly, while ribbonless contacts accounted for almost 50% of all contacts between B12 and the identified ganglion cell, we did not observe this type of synaptic contact between B6a/B6b bipolar cells and their postsynaptic ganglion cell.

## Discussion

We acquired an electron microscopic dataset from the peripheral European robin retina using ssmSEM to (1) compare the double cone anatomy between two avian species that occupy different ecological niches and migratory lifestyles, (2) expand the analysis of the postsynaptic connectivity of double cones and uncover species-specific bipolar cell type differences, and (3) provide first insights into European robin specific circuits, including the identification of a midget-like circuit, which receives principal member input, and a circuit receiving highly selective accessory member input.

In general, cones in many bird retinas including chicken possess an oil droplet^7,36,50,51^, which acts as long pass filter, absorbing short-wavelength light^35^. As double cones may play a crucial role in light-dependent radical pair-based magnetoreception^13,52^, potentially based on the magnetically sensitive blue light sensor cryptochrome 4^14^ expressed in double cone outer segments^4^, the absence of an oil droplet within the accessory member of double cones in the long-distance migratory European robin would allow blue light to be transmitted to the outer segment, enabling cryptochrome-based magnetoreception. This may represent an adaptation to the bird’s long-distance migration. However, it is unclear at this point whether the ellipsoid body of the accessory member in the European robin contains carotenoids that could absorb blue light as described in other bird species^50^.

For double cones to act as polarised light sensors, a fixed orientation is required either between the members of a double cone or between neighbouring double cones, which, based on known orientations from invertebrates, could be either 90° in two directions or 60° in three directions of polarisation sensitivity^53^. Measuring the orientation of both members within a double cone revealed a relative angle of 119° in the robin and 179° in the chicken. The 179° relative orientation in the chicken would most likely render any comparison of signals originating from the two members of the double cone insensitive to polarised light. In contrast, the 119° relative orientation in the robin could allow for some sensitivity to polarised light, considering the three-dimensional tuning properties of polarisation sensitive neurons in the cricket^54^. The previously described ±180° bimodal distribution of orientations between neighbouring double cones in the peripheral retina^12^ may enable the detection of a magnetic field without variations in polarized light masking the magnetic signal^15^. Using the connecting cilium as a landmark to determine the orientation of the double cone members, we can only speculate whether the other cell compartments have a fixed orientation within the cell and in relation to the connecting cilium. Thus, the angles determined here may not exactly represent the angles of the double cone outer segments towards each other. Additionally, Flamarique and colleagues^10^ suggested that, in some fish species, the contact membrane area between two members of a double cone acts as a dielectric mirror. They proposed that this could facilitate polarisation sensitivity by causing an anisotropic transfer of polarised light to the outer segments, resulting in a detectable change in light intensity. Whether the connecting membrane of double cones in birds also has this property is currently unknown.

Functional experiments in turtles and tiger salamanders suggest that photoreceptors can be electrically and chemically coupled^55–58^. The tight apposition of identified contacts between principal and accessory member telodendria with contacted photoreceptor terminals could suggest the presence of gap junctions, especially compared to the clear synaptic cleft observed between bipolar dendrites and double cone terminals. On the other hand, single cell transcriptomics data indicate that at least one member of the double cone expresses a kainate receptor^59^, indicating chemical synaptic transduction. We observed frequent ribbon synaptic contacts between the telodendria of the accessory member and the terminal of the principal member within the same double cone, resulting in a potential chemical signal transduction from principal members to their intra-double cone accessory members.

We identified 15 different bipolar cell types contacting double cones in the European robin, excluding single cone-selective bipolar cells. We matched the majority of bipolar cell types to previously known ones from the chicken retina, but also found four novel types. Cells that were clustered into each type formed uniform dendritic and axonal field mosaics with a varying extent of overlap (or coverage factor) in their dendritic fields.

An exception to this rule are B6b bipolar cells, in which we only observed a mosaic in its axonal fields. Their unique and specific connectivity results in the dendritic field from one cell interweaving with that of neighbouring cells, thereby obscuring a clear mosaic. While it is too early to speculate on a possible functional role of the peculiar orientation selectivity of B6b bipolar cells, it is unlikely to be coincidental. B6b cells appear to receive accessory member inputs from two different halves/sides of their dendritic fields with nearly 180° opposing orientations, but complete B6b reconstructions would be required for statistical analyses. Furthermore, reconstructed postsynaptic ganglion cells seem to preserve this selectivity. Given the absence of oil droplets in the inner segments of accessory members and the selective wiring of B6b bipolar cells, it is tempting to speculate that this circuit might be involved in processing magnetic or polarised light information. However, lacking functional information, it is unclear at this point how the dendrites of B6b cells could integrate such information.

To date only one midget-like bipolar cell type was identified in chicken (B5a in ref. ^7^) and pigeon (B8 in ref. ^19^). Surprisingly, for a peripheral area within the retina, we found four bipolar cell types in the European robin that exhibit midget-like characteristics, namely B11, B12, B4b and B5, that all innervate principal member terminals, suggesting four independent midget-like pathways. In the central primate retina, midget bipolar cells receive synapses from one single cone and transmit their signal to one ganglion cell, mediating high acuity vision^60^. In the peripheral primate retina, several midget bipolar cells converge onto one ganglion cell and also receive synapses from several cones within their dendritic fields^61,62^. Since B12 cells in our dataset exhibit the strongest midget-like character, we analysed postsynaptic ganglion cells and identified a B12-selective ganglion cell that receives specific input from at least 18 B12 bipolar cells, confirming the existence of at least one midget-specific circuit in a bird retina. These results raise the question of whether European robins have a greater demand for high-acuity peripheral vision than chickens since cells B12 and B11 were not identified in the chicken retina. Additionally, we found a midget-like system receiving input from the principal member of a double cone, rather than single cones, but this finding may be consistent with the proposed involvement of double cones in high-acuity vision^9^. Since this dataset was collected from the periphery of the retina, one pressing question is whether birds also have one or even several midget systems near the fovea to enable high acuity vision, in which case one ganglion cell may receive input from only one bipolar cell. Findings from a comprehensive single-cell transcriptomic analysis that comparing retinal neurons across vertebrates^17^ indicate that chickens have four orthotypes of midget-like bipolar cells. Morphologically, we previously identified only one midget-like bipolar cell type in the chicken, whereas four European robin types were found in the present study. These four types may correspond to the four clusters identified in the transcriptomic data. Whether chickens possess more midget-like bipolar cells in other retinal regions is open for future work.

Single cell transcriptomic data from the chicken retina provide detailed information about the number of bipolar cell types, including evidence of ON or OFF response properties for each cell type^59^. Upon comparing the identified morphologies of specific clusters with our data, B3 cells (corresponding to cluster 6) and, surprisingly, B2 cells (cluster 12), the cell type with the highest number of ribbon synaptic contacts typically associated with ON responses, are most likely OFF bipolar cells. The stratification profiles we quantified in the European robin (Supplementary Figs. 3 and 4) can be compared with previously described immunohistochemical ON bipolar cell markers such as EGFR and PKCα^26^. The EGFR labelling pattern best matches the stratification of B4a bipolar cells. However, Balaji et al., 2023 found that EGFR positive bipolar cells comprise a subtype of HCN1 positive bipolar cells, indicating that the EGFR labelled cells are at least two types of bipolar cells. Unfortunately, due to additional labelling of amacrine cells in the HCN1 signal and only a weak EGFR staining of this subtype, we were not able to match the second bipolar cell type. The PKCα labelling pattern^26^ could be best explained by the profile from B4b bipolar cells having a broader middle stratification band than the upper and lower stratifications (Supplementary Fig. 3). The soma position of B4b bipolar cells also matches the lowest layers of somas in the PKCα staining indicating that the PKCα staining is comprised of multiple cell types, as was suggested before^26^. A potential second cell type that might be included in the PKCα signal is the monostratified B11, as it stratifies only slightly above the broad 2^nd^ B4b stratum and therefore would not appear as a separate band in the fluorescent staining (Supplementary Fig. 3). We, therefore, conclude that B4a, B4b, and possibly B11 bipolar cells are subtypes of the EGFR and PKCα signal and therefore are good candidates for ON bipolar cells.

Overall, we found numerous species-specific differences in the anatomy and downstream connectivity of double cones in the chicken and robin retinas that likely reflect adaptations to their visual requirements and behaviour. Nevertheless, future investigations are needed to further explore the functional consequences of these structural differences and explore potential regional specializations within the retina.

### Supplementary information

Supplementary Information

Description of Additional Supplementary Files

Supplementary Data 1

Reporting Summary

## Data Availability

The raw aligned dataset is available in webknossos under: https://webknossos.mpinb.mpg.de/links/1GsNiZcF0C3fJjJC. Additionally, bipolar or photoreceptor cell skeletons, synapses or other raw data are provided in this repository: 10.17617/3.Z0HMMH^63^.

## Code availability

Analysis of skeletons and synapse annotations was performed using Matlab2023b. Statistics on double cone orientations were performed with a custom written R script from^31–33^. All code is available in the data repository^63^.
