# Supplementary material for: Species–specific circuitry of double cone photoreceptors in two avian retinas
Source: Commun Biol. 2024 Aug 14;7:992. doi: 10.1038/s42003-024-06697-2 (PMC11325025; doi:10.1038/s42003-024-06697-2)
Supplement: Supplementary file 1 — Supplementary Information [file 42003_2024_6697_MOESM1_ESM.pdf]

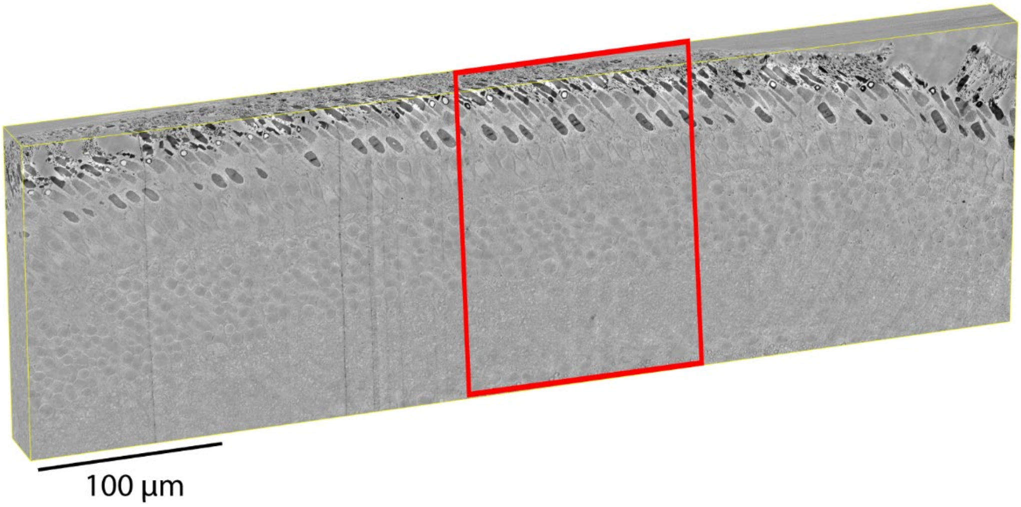

Suppl. Fig. 1 EM volume of a retinal piece from the European robin located in the dorsal periphery of the retina. Area where bipolar cells were reconstructed is highlighted with red rectangle.

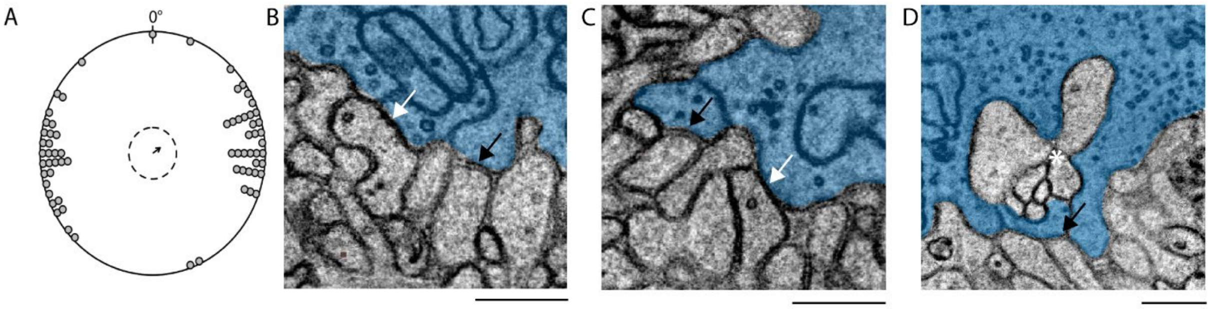

Suppl. Fig. 2 Overall double cone orientation and differential photoreceptor and bipolar cell contacts to photoreceptor terminals. (A) Angles of complete double cones within the dataset of the European robin (N=67). Grey dots represent orientation of one double cone within the dataset in 5° bins. The arrow displays the mean orientation of all double cones. The arrow length represents the directedness based on their Rayleigh values (r value). **(B-D)** Photoreceptor terminal highlighted in blue. Basal contacts between bipolar cell dendrites and photoreceptor terminals form a cleft where outer membranes from dendrite and photoreceptor are visibly separated (black arrow). Membranes from photoreceptor telodendria appear more heavily stained and at basal contacts between photoreceptor telodendria and photoreceptor terminals, membranes appear as one membrane (white arrows). Photoreceptor telodendria also contact terminal as central elements of a ribbon synapse (white asterisk in D). All scale bars = 500 nm.

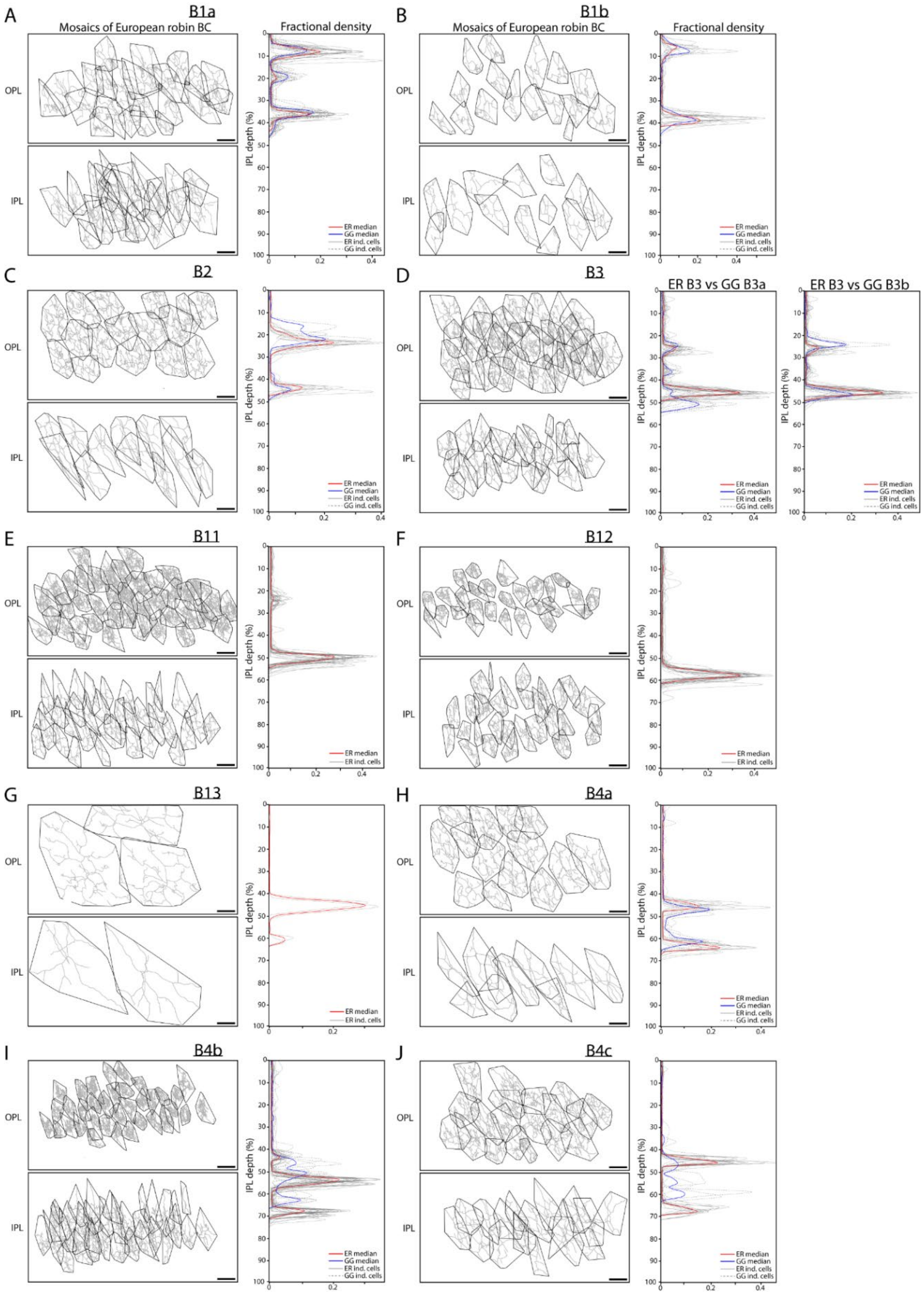

Suppl. Fig. 3 Dendritic (upper left panel) and axonal field (lower left panel) mosaics from European robin retina and plot profiles from the axonal stratification depth (right panel) in European robin (red line) and chicken (blue line) for bipolar cell type B1a **(A)** to B4c **(G)**. In case of multi-stratification, the IPL mosaics include all stratifications. BC = bipolar cell, ER= European robin, GG= chicken, OPL = outer plexiform layer, IPL = inner plexiform layer.

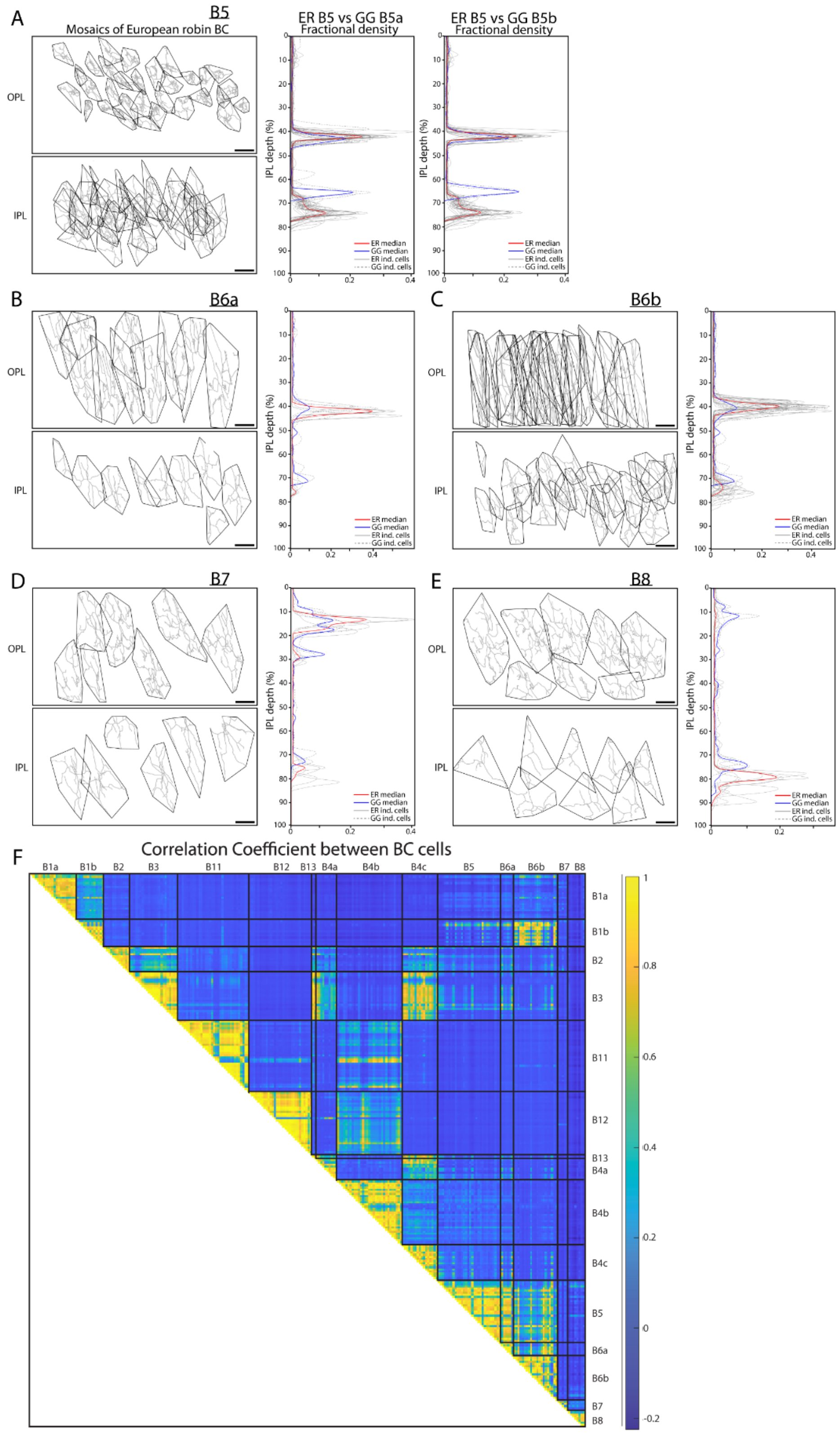

Suppl. Fig. 4 Dendritic (upper left panel) and axonal field (lower left panel) mosaics from European robin retina and plot profiles from the axonal stratification depth (right panel) in European robin and chicken for bipolar cell type B5 **(A)** to B8 **(E)**. In case of multi-stratification, the IPL mosaics include all stratifications. **(F)** Similarity matrix based on correlation coefficients between IPL stratification profiles of individual cells. High numbers indicate a high similarity between two cells. BC = bipolar cell, ER= European robin, GG= chicken, OPL = outer plexiform layer, IPL = inner plexiform layer.

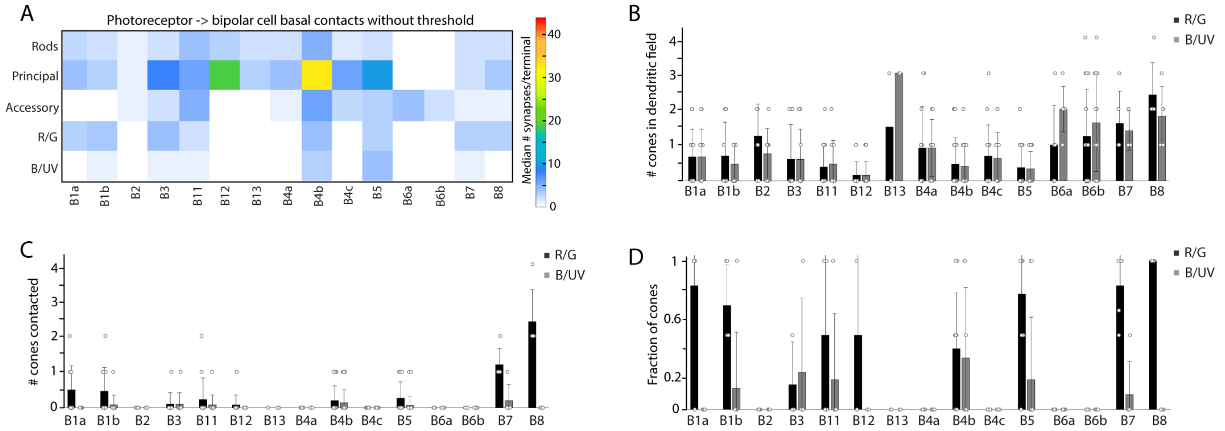

Suppl. Fig. 5 Additional quantification of European robin bipolar cell to photoreceptors connectivity. (A) Connectivity matrix with median number of basal contacts per bipolar cell type without thresholding. **(B)** Mean number of R/G and B/UV single cone terminals within dendritic field of different bipolar cells. **(C)** Mean number of contacted R/G or B/UV single cone terminals for different bipolar cell types. **(D)** Fraction of contacted/in dendritic field R/G or B/UV single cones for different bipolar cell types. R/G = red and green single cones, B/UV = blue and ultraviolet single cones.

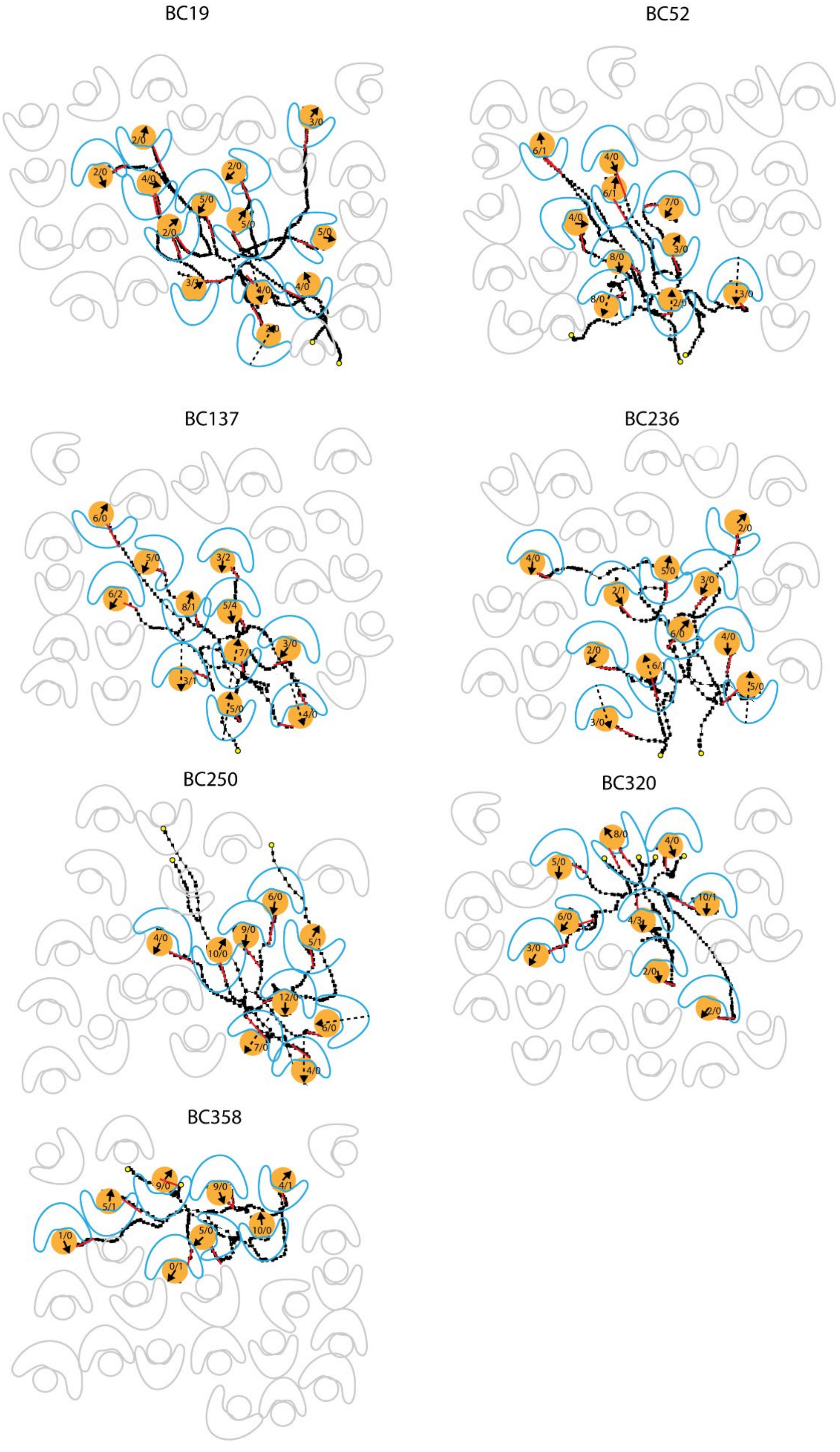

Suppl. Fig. 6 Individual dendritic fields of B6a cells as in Fig. 4A. Numbers of individual basal/ribbon contacts are indicated in the individual accessory member terminals.

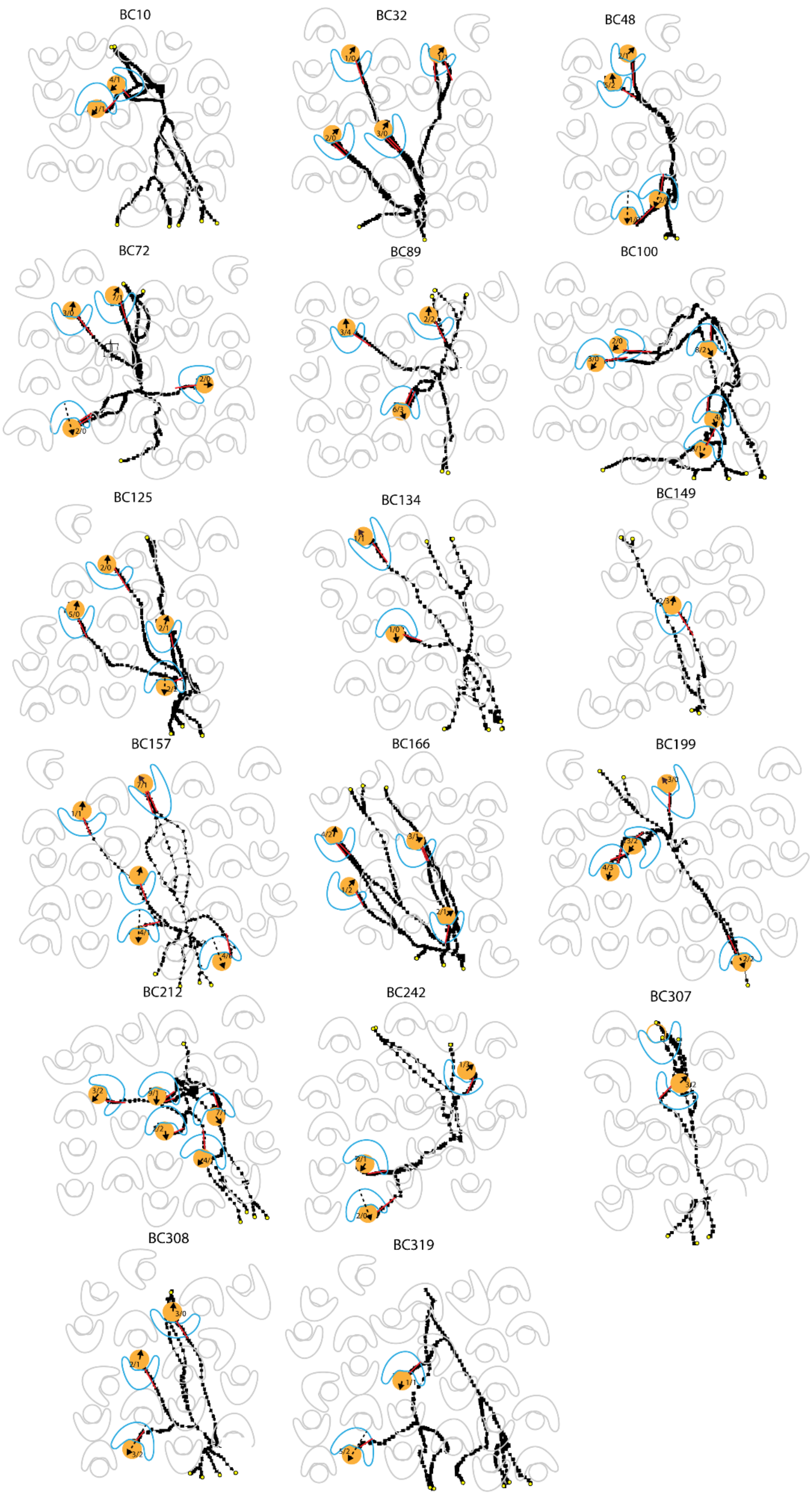

Suppl. Fig. 7 Individual dendritic fields of B6b cells as in Fig. 4B. Numbers of individual basal/ribbon contacts are indicated in the individual accessory member terminals.
