# Supplementary material for: Species–specific circuitry of double cone photoreceptors in two avian retinas
Source: Commun Biol. 2024 Aug 14;7:992. doi: 10.1038/s42003-024-06697-2 (PMC11325025; doi:10.1038/s42003-024-06697-2)
Supplement: Supplementary file 2 — Description of Additional Supplementary Files [file 42003_2024_6697_MOESM2_ESM.pdf]

Description of Additional Supplementary Files

File name: Supplementary Data 1

Description: Tif-Stack showing the inner segments of a principal and accessory member of one double cone in the European robin retina. Oil droplet from principal member marked with # on slice 1. To track the accessory member backwards the paraboloid body is marked with * on slice 218.
