# Supplementary figures and images for: Species–specific circuitry of double cone photoreceptors in two avian retinas
Source: Commun Biol. 2024 Aug 14;7:992. doi: 10.1038/s42003-024-06697-2 (PMC11325025; doi:10.1038/s42003-024-06697-2)

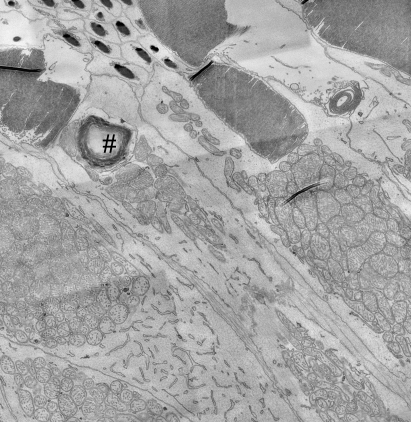

Supplement: Supplementary file 3 — Supplementary Data 1 [file 42003_2024_6697_MOESM3_ESM.tif]
